# Emerging Microbial Concerns in Food Safety and New Control Measures

**DOI:** 10.1155/2014/251512

**Published:** 2014-07-06

**Authors:** Moreno Bondi, Patrizia Messi, Prakash M. Halami, Chrissanthy Papadopoulou, Simona de Niederhausern

**Affiliations:** ^1^Department of Life Sciences, University of Modena and Reggio Emilia, Via Campi 287, 41125 Modena, Italy; ^2^Food Microbiology Department, CSIR-Central Food Technological Research Institute, Mysore 570020, India; ^3^Microbiology Department, Medical School, The University of Ioannina, 451 10 Ioannina, Greece

Food-borne diseases are a widespread and growing public health and economic problem. Recent modifications in food production and processing practices and ever-changing food habits of the consumer are important factors for the incidence of food-borne infections. Many recognized pathogens are of great concern today and new challenges have appeared in the recent years as the role of the interaction between pathogens during infection.

Despite chill chains, chemical preservatives, and a better understanding of microorganisms, food-borne diseases (FBD) represent an important health problem for developed and developing countries. It has been estimated that about 30% of people in industrialized countries suffer from a food-borne disease each year (about 76 million cases of illness, 325,000 hospitalization cases, and as many as 5000 deaths in the United States annually [1]) and 25% of all foods produced globally are lost due to microbial spoilage. The European Food Safety Authority (EFSA) and the European Centers for Disease Control (ECDC), analyzing the information on the occurrence of zoonosis and FBD in 2010 submitted by 27 European Union Member States, reported a total of 5,262 food-borne outbreaks, causing 43,473 human cases, 4,695 hospitalization cases, and 25 deaths. Most of the reported outbreaks were caused by* Salmonella*,* Campylobacter*, bacterial toxins, and viruses. In particular, the scientific report of EFSA and ECDS 2012 [2] shows the decreasing trend in case numbers of a “classic” pathogen as* Salmonella* continues, whereas for other etiological agents of FBD a slight or no decrease (*Listeria monocytogenes* and* Campylobacter*, resp.) or an increase (verotoxigenic* Escherichia coli*) in human cases has been observed. Another common cause of FBD worldwide is* Staphylococcus aureus*, responsible of an estimated 241,000 illnesses per year only in the United States [3].

In this special issue you will find an in-depth analysis on the importance of Staphylococcal food-borne disease. Recent findings of high prevalence of* S. aureus* including MRSA in raw retail meat impose a potential hazard to consumers, both as classic FBD and as a potential source of colonization of food handlers. An emerging problem that involves this field is therefore the increase of multidrug resistance in pathogenic, opportunistic, and spoilage bacteria, which can reach humans through the food chain. In recent years, for example, a new strain of* S. aureus*, livestock-associated methicillin-resistant* Staphylococcus aureus* (LA-MRSA), has been recognized as a novel pathogen that has become a rapidly emerging cause of human infections [4]. As demonstrated by scientific evidence [5–7], genes for resistance may be transferred by mobile bacterial genetic elements frequently together with those encoding for virulence factors, a phenomenon that may lead to the appearance of microorganisms with increased pathogenicity.

This special issue also shows that, in some cases, the same pathogenic microorganisms, as well as representing a direct risk to health, can make the host more receptive to infection by other types of microorganisms, such as food-borne infections by protozoa.* Giardia duodenalis* is often seen as an opportunistic pathogen and one of the major food and waterborne parasites. For example, the* Giardia* coinfection with pathogens like* Vibrio cholerae* and* Rotavirus* seems to play a significant role in the occurrence of this parasite. Some important factors are responsible for the regulation of the diarrheal disease spectrum of a population, as the age distribution of the diarrheal cases that was very much dependent on the coinfection rate of* Giardia* infection.

Outbreak investigations have suggested that a lot of risk factors linked to the raw ingredients, improper handling of cooked or processed food, an inadequate cleaning, and disinfection of equipment used in food processing, are the main source of contamination. For this reason, it is important to have new technologies that make it possible to determine such problem in real time. Here you can find a new method to detect food contamination by sensory analysis of foods. A novel electronic nose (EN) represents the tool that provides this capability. The characteristic flavour of volatile organic compounds (VOCs), the so-called fingerprint, may offer information about safety and quality of food, performing sometimes as an indicator of process mistakes as well [8]. Indeed some volatile compounds can originate from biochemical processes of food as a consequence of technological food chain or product storage. Unwanted smell, the so-called off-flavour, may involve substances originating from the metabolism of spoilage microorganisms, bacteria, and fungi that adulterate naturally or unintentionally the food before or during its production [9]. This EN could represent a rapid mean for controlling and improving the microbiological quality of food and a potential and useful tool for the early detection of microbial growth.

In this special issue a method to determine the actual risk of cereulide toxin production, by* Bacillus cereus*, in different types of food is also reported. Several* B. cereus* strains possess the genetic fittings to produce two different types of toxins, the heat-stable cereulide or different heat-labile proteins with enterotoxigenic potential. Unlike the diarrheal toxins, cereulide is (pre-) formed in food and can cause food-borne intoxication shortly after ingestion of contaminated food. It is possible to use a lux-based real-time monitoring system to assess the significance of the detection of emetic strains and to determine the actual risk of cereulide toxin production in different types of food. Luciferase signals were quantified with software and foods were categorized into three main classes regarding their toxin formation capability: high-risk, risk, and low risk foods. This generalized scheme allows a basic preevaluation of foods and their ingredients concerning their capability to support cereulide formation and should facilitate hazard identification.

Even if the use of refrigeration temperature is the most employed preservative method, spoilage and psychrotrophic pathogens can grow at low temperatures, representing a threatening public health concern and shortening the shelf life of raw foods. Many outbreaks associated with fresh ready to-eat produce have been previously reported due to* E. coli, Listeria,* and* Salmonella* [10]. If bacterial development could be delayed or inhibited, it would be possible to obtain a great advantage relatively to the public health and the shelf life of products. Actually, the consumer preferences are moving towards foods containing lower levels of chemical preservatives, maintaining characteristics of fresh or natural products. Therefore, the preventive measures could be represented by sanitization methods alternative to the use of chemicals. A new microbial challenge in food safety, reported in this special issue, will be the use of physical treatment or the addition of natural preservatives endowed with antimicrobial capability. Recent studies demonstrate the bactericidal efficacy of alternative nonthermal light technologies and their potential as decontamination strategies in the food industry. A range of nonthermal technologies have already been successfully implemented on a number of ready-to-eat fruits and vegetables [11]. Exposure of microorganisms to visible light, particularly at wavelengths of 405 nm, has been shown to be effective in inactivating a range of Gram- positive and Gram-negative bacterial species [12]. Thus, these alternative nonthermal disinfection light techniques could find potential applications for decontamination in the food industry.

The other alternative to chemical food preservatives is the use of lactic acid bacteria (LAB). LAB strains are food-grade organisms that may be used in biopreservation strategies due to their ability to produce several antimicrobial compounds, including organic acids, hydrogen peroxide, and bacteriocins [13]. These microorganisms and their products could be employed as starter cultures [14] and natural preservatives [15] or could be entrapped in a polymeric film for its potential use in the active food packaging field [16]. The study reported in this special issue has provided the first genetic characterization of bioactive* Leuconostoc* spp. isolated from Algerian raw camel milk because of its beneficial effects on human health.* Leuconostoc* spp. plays a crucial role in food biopreservation through the production of bacteriocins with different inhibition spectra and exerts beneficial effects on the microbiological stability and production of aroma compounds in various food products. This study also represents the first report on the application of MALDI TOF MS analysis for the faster and more reliable identification of* L. mesenteroides* based on their low molecular-weight protein profile. The application of MALDI TOF peptide mass fingerprinting has been successfully applied to this bacterial group and has proven to be a simple, quick, and inexpensive complementary method for bacterial identification at the species level.

This editorial collects a brief summary of the topics discussed in the articles that are published in* Emerging Microbial Concerns in Food Safety and New Control Measures*. We hope that readers of this special issue will find some information of interest in order to expand their knowledge in this field and to increase their level of attention on matters here reported.

*Moreno Bondi* *Patrizia Messi* *Prakash M. Halami* *Chrissanthy Papadopoulou* *Simona de Niederhausern*
